# Design and Virtual Workflow of a Patient Database for Clinical and Pharmaceutical Trials Part II: A Prototype for Endocrinology

**DOI:** 10.7759/cureus.30006

**Published:** 2022-10-06

**Authors:** Konstantinos Vezertzis, George I Lambrou, Dimitrios Koutsouris

**Affiliations:** 1 Biomedical Engineering Laboratory, National Technical University of Athens, Athens, GRC; 2 Laboratory for the Research of Musculoskeletal Disorders, National and Kapodistrian University of Athens, Athens, GRC; 3 First Department of Pediatrics, Choremeio Research Laboratory, National and Kapodistrian University of Athens, Athens, GRC; 4 University Research Institute of Maternal and Child Health and Precision Medicine, National and Kapodistrian University of Athens, Athens, GRC

**Keywords:** open-source software, virtual workflow, prototype design, patient recruitment, electronic health record (ehr)

## Abstract

Background

In previous work, we have reported that patient recruitment is closely related to electronic health records (EHR). As a result, the next step of investigation would lead to the implementation of research practices for using EHR in selecting patients for clinical trials. Towards that end, open-source software offers several integrated solutions that can meet the needs of an EHR and patient recruitment.

Aim

In the present work, we have designed a prototype of a patient recruitment system using open-source tools. The proposed prototype can draw data from a patient management system and present selected patients based on specific criteria.

Methods

For the objective of the present study, we have used the methodology described previously. In particular, we recorded numerous integrated solutions for EHR from the area of free and open-source software. Open Electronic Medical Records (OpenEMR) ranked first for functionality and second for usability efficiency. Hence, we relied on OpenEMR to design a prototype patient recruitment system. After the installation and commissioning of OpenEMR, we created appropriate test scenarios. Therefore, populated appropriate patient data in OpenEMR. PhpMyAdmin was installed and commissioned along with the OpenEMR installation. This tool is used to manage MySQL database systems. MySQL is the database system that programmers rely on to develop OpenEMR.

Results

A prototype patient recruitment system was designed, which draws data from a view of the OpenEMR database to present results based on criteria.

Conclusions

After the adaptation of the database and the design of the proposed solution, we concluded, based on the prototype results, that there is potential for developing an integrated patient recruitment management system. This management system can be based on the implementation of complex criteria and present results according to the needs of the end-user.

## Introduction

The electronic medical record (EMR) is a modern reality that is necessary for the information society, and as such, EMR has become a necessity in day-by-day praxis. The EMR is something that, although it does not have a clear definition, everyone knows what it means, even in theory. It is the means of communication between doctor and patient. It includes referrals and test results, recording the course of patients' disease, imaging tests such as X-rays, CT scans, etc., cardiograms, and financial data such as any payments from the user to the medical staff. The EMR serves several different purposes [1, 2]. These purposes include a) practically, it is the primary means of communication for the users involved, i.e., the health professionals, paramedics, and patients. Any information concerning patients, related to their condition such as diagnoses, treatments, instructions to be followed, can be transmitted directly and quickly to the respective users through the use of the medical file, b) in addition, due to the health practitioners' ability to store large amounts of data, the access to a patient's information is immediate, providing a fast and accurate update for the patient's condition, the course of the disease etc., c) further on, it is informally a means of recording personal views, ideas and ways of dealing with illnesses and conditions, separately, d) thus, the medical file is the "storage space" of all necessary data (including clinical and anthropometric) needed for future use, but also for research purposes, such as epidemiological studies, in vivo and in vitro research, evaluation of the quality of the provided services etc., e) also, due to its easy accessibility, it facilitates the immediate recall of data in cases of medical errors, and thus the whole procedure followed during a treatment can be monitored. Therefore, it is feasible to thoroughly monitor the complete course of a patient from diagnosis to therapy and follow-up. In addition, it is easier to prevent future pitfalls or mistakes based on the information at hand, f) in addition, it can be used as a means of exchanging financial data with users; for example, insurance companies can collect the necessary data to decide on the financial coverage of the patient's medical care and g) the medical record is not exclusively a tool of clinical administration or monitoring, but it can also consist of an instrument for research at a deeper level and study to a greater extent. The design of such a medical database depends largely on various other (external) factors, such as local legislation, the population's customs and traditions, the local socioeconomic conditions, the logistical infrastructure, etc. Although such an approach includes a plethora of technical details and programming code, it can still be used in principle as a guide for the clinician to realize that EMR can be a tremendously valuable tool in the daily praxis. As discussed further on, EMR can be a mandatory informational tool for clinics facilitating services otherwise impossible without an electronic data management system. The objective of the present study was to study the design and virtual workflow of a prototype patient recruitment system using free and open-source software, focusing on three types of thyroid gland conditions: primary hypothyroidism, hyperparathyroidism, and nodular goiter. This prototype can draw data from a patient management system and present selected patients based on specific criteria while at the same time ensuring patient privacy and confidentiality. For simplicity, we mention the objective of design and virtual workflow in the present study as design.

A short introduction of endocrinological conditions

Hypothyroidism

The thyroid is a small "butterfly-shaped" gland at the front of the neck. Hypothyroidism is "a syndrome that results from abnormally low secretion of thyroid hormones from the thyroid gland, leading to a decrease in basal metabolic rate. In its most severe form, there is an accumulation of mucopolysaccharides in the skin and edema, known as myxedema. It may be primary or secondary due to other pituitary diseases or hypothalamic dysfunction (MeSH term D007037). The thyroid hormones control how the human body uses energy. Therefore, it affects almost every human organ. The lack of thyroid hormone causes a decrease in bodily functions. The causes of hypothyroidism are still under intense investigation. However, it is known to be caused by inadequate functioning of the gland, inadequate stimulation by the pituitary hormone, and insufficient production of thyrotropin-releasing hormones from the hypothalamus of the brain. There are many reasons why the cells of the thyroid gland cannot produce sufficient hormones. Some of these include the presence of autoimmune diseases, surgical removal of either part or all of the thyroid gland, radiotherapy, congenital hypothyroidism, thyroiditis, medications, lack or excess of iodine, pituitary damage, and rare thyroid disorders [3].

The symptoms of hypothyroidism vary, depending on the severity of the hormone deficiency. When hormone levels are too low, the body cells do not receive enough thyroid hormone and the body's processes begin to slow down. Usually, symptoms evolve gradually and are not immediately noticeable even for years. Many symptoms of hypothyroidism are similar to those of other diseases, so it can easily be confused with another disease. Symptoms of hypothyroidism may include: fatigue, increased sensitivity to cold, constipation, dry skin [3], weight gain, swollen face, hoarse voice, muscular weakness, muscle aches, tenderness and stiffness, increased cholesterol in blood, pain, stiffness or swelling of joints, heavier or irregular menstrual periods, hair thinning, decreased heart rate, and enlarged thyroid gland (goiter). Older patients with hypothyroidism may experience memory problems and depression. Children may experience retardation of physical and mental development [3]. Very young people may start puberty earlier than usual.

The diagnosis of hypothyroidism is based on symptoms and results of blood tests called thyroid function test, which measures the level of thyroid-stimulating hormone (TSH) and thyroxine levels (Τ4). Low T4 levels combined with a high level of TSH indicate hypothyroidism. This is because the pituitary gland produces more TSH to stimulate the thyroid gland to produce more T4. Blood test results showing an increased rate of TSH but normal T4 levels indicate an increased risk of hypothyroidism in the future [4].

The thyroid function test consists of a group of blood tests used to diagnose thyroid problems. Thyroid hormone levels measured in the blood sample are compared with reference ranges. Reference ranges aim to represent the range of measurements found in 95% of the total healthy population. During examining the results of thyroid function tests, it is essential to check the reference ranges, particularly for free thyroxine (FT4), as it is common to differ among labs. Concerning the present study, we considered the reference ranges proposed by the Health Services of Alberta [5].

Hyperparathyroidism

Hyperparathyroidism is a disease in which the parathyroid glands produce excessive parathyroid hormone (PTH) in the bloodstream. It is termed as the "abnormal elevation of the PTH secretion as a response to hypocalcemia. It is caused by chronic kidney failure or other abnormalities in the controls of bone and mineral metabolism, leading to various bone diseases, such as renal osteodystrophy" (MeSH term D006962). The parathyroid glands are located behind the thyroid gland in the lower part of the neck. They are approximately the size of a grain of rice. The parathyroid glands produce PTH, which helps sustain the balance of calcium in the bloodstream and tissues that depend on calcium for proper function [6].

There are two types of hyperparathyroidism. In primary hyperparathyroidism, the enlargement of one or more parathyroid glands causes higher than normal hormone production. In turn, higher hormone production causes high levels of calcium in the blood, resulting in various health problems. Surgery is the most common treatment for primary hyperparathyroidism [6]. Secondary hyperparathyroidism is caused by other diseases or dysfunctions. Secondary hyperparathyroidism causes hypokalemia. In turn, parathyroid glands are charged with increasing the level of calcium in the blood and releasing more parathyroid hormone. Consequently, blood tests show high levels of PTH and low levels of calcium in the blood.

Factors that may contribute to the development of secondary hyperparathyroidism include a) severe calcium deficiency, where due to poor absorption by the digestive system, the human body may not receive enough calcium from the diet, b) severe vitamin D deficiency. Vitamin D aids in maintaining adequate calcium levels in the blood. It also helps the digestive system to absorb calcium from food. The human body produces vitamin D during skin exposure to sunlight. Therefore, some vitamin D is also consumed in food. If the vitamin D level is low, then the calcium level may get decreased, and c) chronic kidney failure. The kidneys convert vitamin D into a form that the body can utilize. If the kidneys function poorly, then usable vitamin D may be reduced. By extension, the calcium level falls, resulting in an increase in the level of PTH. Chronic kidney failure is one of the most common causes of secondary hyperparathyroidism [7, 8], and d) Crohn's disease. A serious bowel condition, such as Crohn's disease, may cause calcium absorption problems from food into the blood, resulting in consistently low calcium levels [9].

Usually, hyperparathyroidism causes few or no symptoms at all. The severity of symptoms is not always associated with the calcium level in the blood. Individuals with mildly elevated calcium levels may experience symptoms. In contrast, individuals with high calcium levels may have few or no symptoms. Symptoms can be broad and include depression, fatigue, thirst, frequent urination, feeling unwell, loss of appetite, muscular weakness, constipation, abdominal pain, loss of concentration, and mild confusion. If left untreated, high blood calcium levels may cause vomiting, drowsiness, dehydration, severe confusion, muscle spasms, bone pain or tenderness, joint pains, cardiac arrhythmia, and hypertension. Also, it may cause several other serious complications, such as osteoporosis, bone fractures, kidney stones, kidney obstruction, kidney damage or insufficiency, peptic ulcers, and pancreatitis. In very severe cases of hyperparathyroidism, high calcium levels may lead to rapid kidney failure, loss of conscience, coma, or life-threatening heart rhythm abnormalities [9].

It is important to diagnose secondary hyperparathyroidism as soon as possible. Without treatment, it may gradually get worse and lead to complications. Nevertheless, in most cases, the condition is mild to moderate and remains stable for years. The diagnosis of hyperparathyroidism is made after a blood test that shows the following: a) high levels of parathyroid hormone, b) high levels of calcium in the blood, usually with low levels of phosphorus, c) DEXA scan (bone density scan) can help diagnose bone loss, fractures, or bone softening, while X-rays, CT scans or ultrasounds may reveal calcium deposits or kidney stones [10].

A PTH blood test measures the level of PTH in the blood. This test is used to help diagnose hyperparathyroidism, the cause of abnormal calcium levels, or to control chronic kidney disease. PTH controls the levels of calcium and phosphorus in the blood. The PTH is produced by the parathyroid glands, four pea-sized glands located behind the thyroid gland. If blood calcium levels are low, the parathyroid glands release more hormones. As a result, the bones release more calcium in the blood, and the amount of calcium released by the kidneys in the urine decreases. Also, vitamin D is converted to a more reactive form, causing the bowels to absorb more calcium and phosphorus. If the calcium level is too high, parathyroid glands release less hormone, and the whole process is reversed. PTH levels that are either too high or too low may cause kidney and bone problems, as well as changes in vitamin D and calcium levels. Tests for calcium and phosphorus levels in the blood can be made simultaneously with PTH blood tests. Bone density X-rays measure the density of minerals, such as calcium, in bone. This information helps health professionals to assess bone strength. Calcium and vitamin D are essential if bone density is lower than normal. Concerning the present study, we consider the reference ranges proposed by the Health Services of Alberta [10].

Nodular Goiter

Nodular goiter "is an enlarged thyroid gland containing multiple nodules (thyroid nodules), usually resulting from recurrent thyroid hyperplasia and involution over many years to produce the irregular enlargement. Multinodular goiters may be nontoxic or may induce thyrotoxicosis" (MeSH term D006044). Nodular goiter can be divided into diffuse goiter, where the entire thyroid gland swells and gives a soft feel to the touch, and nodular goiter, which consists of solid or fluid-filled lumps called nodules formed within the thyroid, giving the feeling of a lump at the touch of the thyroid gland. Nodules may be single or multiple. Nodular goiters are common and endemic in areas with low iodine concentration in drinking water, and most nodules are benign.

Goiter can have many possible causes, such as a) hyperthyroidism, b) hypothyroidism, c) hormonal changes during puberty, pregnancy, or menopause, d) not enough iodine in the diet, e) taking certain medications, such as lithium, used to treat certain mental health conditions, f) thyroiditis, g) radiation therapy to the neck or chest, such as radiation therapy for neck cancer, h) nodules or cysts within the thyroid, most are harmless, but their condition should be evaluated, and i) thyroid cancer. Goiter is age- and gender-independent, but the chances increase with age [11]. Women are also more likely to develop goiter [11].

The size of a goiter may vary between patients. In most cases, the swelling caused by a goiter is small and shows no symptoms. In more severe cases, symptoms may include the following a) cough, b) tightening in the neck area, c) alterations in the voice, such as hoarseness, d) dysphagia, and e) breathing difficulty. The diagnosis of goiter is usually made during a physical examination when thyroid swelling is detected. The presence of goiter indicates that the thyroid gland is abnormal. Therefore, it is essential to determine the cause of the goiter. As a first step, a thyroid function test is performed to determine if the thyroid is inactive or excessive. Any subsequent examinations will depend on the results of the thyroid examination. Other tests that help diagnose nodular goiter may include thyroid ultrasound or fine-needle aspiration biopsy [12].

Ultrasound is a test that uses reflected sound waves to produce an image of organs and other structures in the body. Ultrasound does not use X-rays or other forms of harmful radiation. Ultrasound is more helpful in examining organs and structures which are uniform and solid or filled with liquid. Fine-needle aspiration biopsy is a method of collecting cells from the thyroid gland to control cancer cells, infections, or other diseases [12].

## Materials and methods

Software

In a previous report, we recorded numerous integrated solutions for EMR from the field of free and open-source software [13]. The list of available integrated solutions is summarized in Table 1. In Table 1, the columns "Active Maintainer," "Date of the Last Update," "Accessed," and "Comments" were added. The active maintainer column lists the manufacturers or developers who have developed their solutions and are actively maintaining them.

The column of "Date of Last Update" refers to the date of the last update of the EMR software. The date is derived from a) the system's websites, b) sourceforge.net [14], and c) github [15]. The update is proposed to have occurred within six months from the date of finalization of Table 1. The "Accessed" column records the date of access to the websites and portals related to the EMR software. Further on, in the "Comments" column, comments are recorded regarding checking the date the websites and portals were accessed. The following short list was compiled based on the data collection carried out, according to the columns as mentioned earlier.

**Table 1:** Indicative list of Free/Libre Open Source Software (FLOSS) healthcare systems.

No.	Title of EHR	URL	Active maintainer	Date of last update *	Accessed	Comments
1	Bahmni	https://www.bahmni.org/	Bahmni Coalition	30/10/2019	8/11/2019	
2	Care2X	http://www.care2x.org/	Care2x Team	29/11/2015	15/11/2019	
3	DHIS2	https://www.dhis2.org/	University of Oslo	3/10/2019	15/11/2019	
4	ElixirAid HIS	http://elixiraid.com/	Gescis technologies ltd.	29/9/2016	15/11/2019	
5	ERPNext based HIS	https://erpnext.com/healthcare	Frappé Technologies	22/7/2019	15/11/2019	This is an ERP system with some health modules
6	FreeMED	https://freemedsoftware.org/	FreeMED Software Foundation	4/11/2013	15/11/2019	
7	GNU Health	http://health.gnu.org/home	GNU Solidario	11/11/2019	15/11/2019	
8	GNUmed Project	http://wiki.gnumed.de/bin/view/Gnumed/WebHome	N/A	31/8/2017	15/11/2019	
9	HospitalOS	http://www.hospital-os.com/th/	Thailand Research Fund	24/10/2018	15/11/2019	
10	HospitalRun	http://hospitalrun.io/	HospitalRun	18/5/2017	15/11/2019	
11	LibreHealth EHR	https://librehealth.io/	Tony Mc Cormick	19/11/2018	15/11/2019	
12	NOSH	https://noshemr.wordpress.com/	Michael Chen, MD	14/8/2018	15/11/2019	
13	Odoo ERP and HIS modules	https://apps.odoo.com/apps/modules/category/Medical/browse	Odoo	N/A	15/11/2019	This is an open-source ERP, with commercial HIS modules
14	One Touch EMR	https://www.onetouchemr.com/	Ν/Α	Ν/Α	15/11/2019	Commercial application
15	Open Hospital	https://www.open-hospital.org/en/	INFORMATICI SENSA FRONITERE	15/10/2019	15/11/2019	
16	OpenEHR	https://www.openehr.org/	Ν/Α	Ν/Α	15/11/2019	It is technology, specifications, standards. Applications are based on the OpenEHR standard.
17	OpenEMR	https://www.open-emr.org/	OpenEMR Foundation	15/11/2019	15/11/2019	
18	OpenMAXIMS	http://www.imsmaxims.com/solutions/opensource/	IMS MAXIMS	6/6/2017	15/11/2019	
19	OpenMRS	https://openmrs.org/	OpenMRS Inc.	30/4/2019	15/11/2019	
20	OSEHRA	https://www.osehra.org/	N/A	N/A	15/11/2019	It is a community, not an application
21	Solismed	https://www.solismed.com/	Intesync LLC	5/10/2017	15/11/2019	
22	WorldVistA	http://worldvista.org/	US Dept. of Veterans Affairs	30/11/2018	15/11/2019	
23	HOSxP	http://www.hosxp.net/joomla25/	Bangkok Medical SW	17/4/2013	15/11/2019	
24	OSCAR	http://oscar-emr.com/	Mc Master University	16/2/2016	15/11/2019	
25	THIRRA	https://www.medfloss.org/node/421	THIRRA HER Sys.	31/1/2017	15/11/2019	
26	ZEPRS	https://en.wikipedia.org/wiki/ZEPRS	RTI	4/11/2014	15/11/2019	
27	ClearHealth	http://clear-health.com/	N/A	N/A	15/11/2019	It has been acquired and operates as a commercial application
28	MedinTux	https://medintux.org/	Dr. Roland Sevin	19/6/2016	15/11/2019	

Next, the final choice emerges after reviewing articles and descriptions related to the systems, either from their sites, or from third party sites. In particular:

Bahmni

Bahmni is a health information system that combines and extends existing open-source applications to a single integrated solution [16]. Bahmni EMR subsystem is based on OpenMRS in the backend, while the front end is developed on Javascript. Therefore, if the Bahmni proposal ended up as the final solution for the EMR system, it would be equivalent to choosing OpenMRS. So, we conclude that for simplicity reasons, the choice of Bahmni is not preferred for the design of our patient recruitment prototype.

DHIS2

The system's full name is District Health Information Software 2 [17]. It is an open-source, web-based system that offers functions of data warehousing, data visualization, and real-time data analysis. DHIS2 interoperates with more than 60 applications as a data warehouse for tasks such as quality control and scorecards. So, we conclude that DHIS2 offers different functionality than an EHR system. Thus, it is not preferred for the design of our patient recruitment prototype.

GNU Health

GNU Health [18] is a free/open-source software project for health professionals, health facilities, and government agencies. Its modular design allows its use, from small private clinics to large national health systems. It provides subsystems such as EMR, Hospital Management (HMIS), and Health Information System (HIS). This system provides more functionality than is needed for the purposes of the present study. So, we conclude that for simplicity reasons, the choice of GNU Health is not preferred for the design of our patient recruitment prototype.

Open Hospital

Open Hospital [19] is a system that offers similar functionality such as GNU Health. It is a free/open-source software suitable for managing health units and non-governmental organizations in developing countries. This system provides more functionality than is needed for the purposes of the present study. So, we conclude that for simplicity reasons, the choice of Open Hospital is not preferred for the design of our patient recruitment prototype.

OpenEMR

OpenEMR [20] is an open-source system that provides EHRs and medical practice management solutions. It is certified by the Office of the National Coordinator for Health IT (ONC). The ONC states, "Health information technology (Health IT) makes it possible for health care providers to better manage patient care through secure use and sharing of health information. Health IT includes the use of EHRs instead of paper medical records to maintain people's health information". One of the features of OpenEMR is multilingual support, including Greek.

OpenMRS

Similarly, OpenMRS [21] is an open-source platform that allows the design of customized medical record systems. Implementations of OpenMRS exist mainly in developing countries and is supported by the Regenstrief Institute and the philanthropic health organization "Partners in Health."

OpenEHR

OpenEHR [22] is not a software but the technology name for eHealth, which includes open specifications, clinical models, and software that can be used to create standards and build information and interoperability solutions for healthcare. EHRServer and EtherCIS [23] platforms are open-source applications based on openEHR. Summarizing those mentioned above, we obtained Table 2 with EMR applications, platforms, and subsystems, which are candidates for the design of the patient recruitment prototype.

**Table 2:** Candidate tools for the design of the patient database.

No.	Title of her	URL
1.	OpenEMR	https://www.open-emr.org/
2.	OpenMRS	https://openmrs.org/
3a.	EHRServer (based on openEHR)	https://github.com/ppazos/cabolabs/ehrserver
3b.	EtherCIS (based on openEHR)	https://ethercis.org/

**Figure 1:**
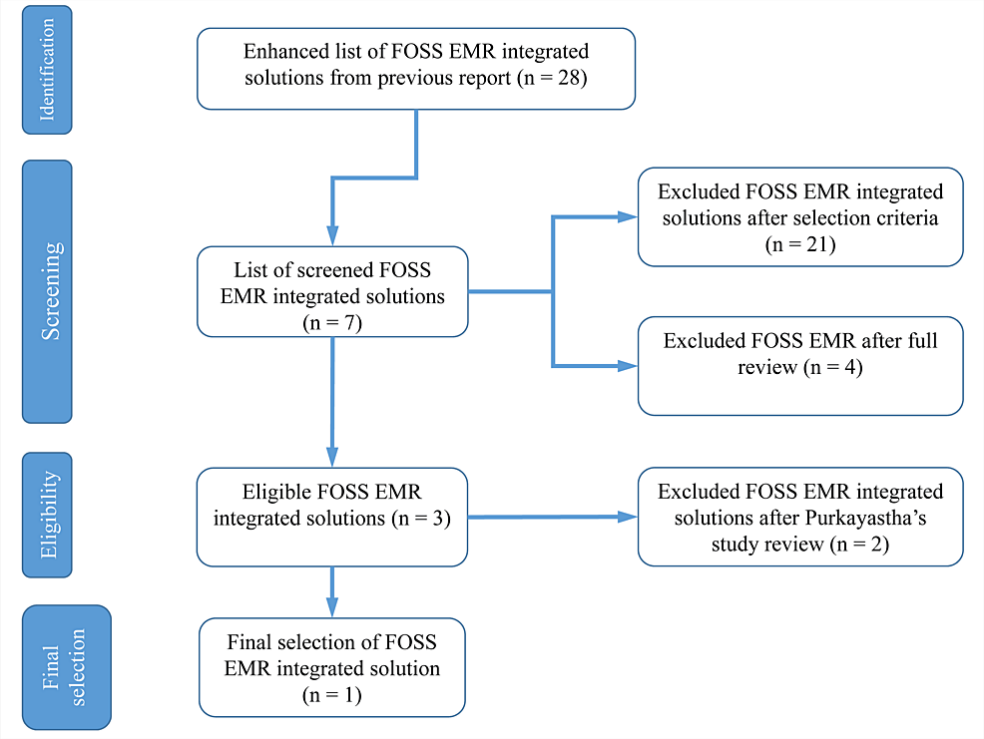
Selection flow chart of development tools.

The enlisted tools in Table 2 have also been previously proposed by Purkayastha S et al. (2019) as an optimal combination for developing health-related applications [24]. Their study reported the aforementioned tools as optimal, regarding their functionality and efficiency of use, offered by open source EHR systems. More specifically, their study concluded that the five systems with the top world ranking in the specific area are OpenEMR, OSEHRA Vista, OpenMRS, GNU Health, and OpenEHR. From these, OpenEMR ranked first for functionality and second for efficiency in its use. Hence, we relied on OpenEMR for the design of the patient recruitment prototype. The flowchart of the selection is presented in Figure 1.

Methodology

The installation, configuration, and commissioning of OpenEMR took place based on the instructions presented on the technology website TechRepublic. Additionally, the installation, configuration, and commissioning of phpMyAdmin took place according to the instructions presented on the technology websites linux4one and TechRepublic. At the time of writing this study, the openEMR version was 5.0.2, while the phpMyAdmin version was 5.0.1. The version of phpMyAdmin, which was installed according to the instructions presented on the above-mentioned technology websites, was 4.6.6. The upgrade to the final version, 5.0.1, was conducted with the assistance of the technology website DevAnswers.

The installation, configuration, and commissioning of OpenEMR were successfully implemented on both a workstation (laptop) and virtual machines developed on cloud servers. More specifically, OpenEMR was installed on IaaS Okeanos of GRNET [25]. For security and stability reasons, the work was carried out in installing the okeanos system. The virtual machine created for the purpose of the present study is based on Ubuntu Server 16.04 operating system, with a dual-core processor, 8 GB RAM memory, and 60 GB storage space. Also, we used a workstation on which the USBWebserver v8.6.5 package was installed to design the prototype form that presents the results after processing [14]. In addition, we have used two different approaches for the database's backup. First, the database was kept in external storage running locally on the server. The second was performed manually through the phpAdmin system by exporting the database to external storage.

After the installation and commissioning of the necessary software for our study, we needed to create the appropriate control scenarios. Thus, we should populate suitable patient data on OpenEMR. Patient data can be obtained in a variety of ways. For example, we may use tools to create dummy patient data, such as synthea or mockaroo. Moreover, we may use open data from specialized web portals, such as the European Union's open health data web portal.

To avoid the violation of sensitive personal data, the solution of creating dummy patients is preferred. For the sake of convenience, dummy patients are referred to as "patients." Patient design and creation consider some specific features. Patients in our study experience diseases associated with the thyroid gland. We will focus on two types of thyroid gland diseases and one parathyroid gland disease: hypothyroidism, nodular goiter, and secondary hyperparathyroidism. For each disease, it is examined whether the values of the indicators are affected by gender, age, and weight. Accordingly, groups of patients are created based on the values that indicate pathological or physiological conditions. We aim to suggest an appropriate model for reliable patient recruitment according to the appropriate decision-making criteria.

**Figure 2:**
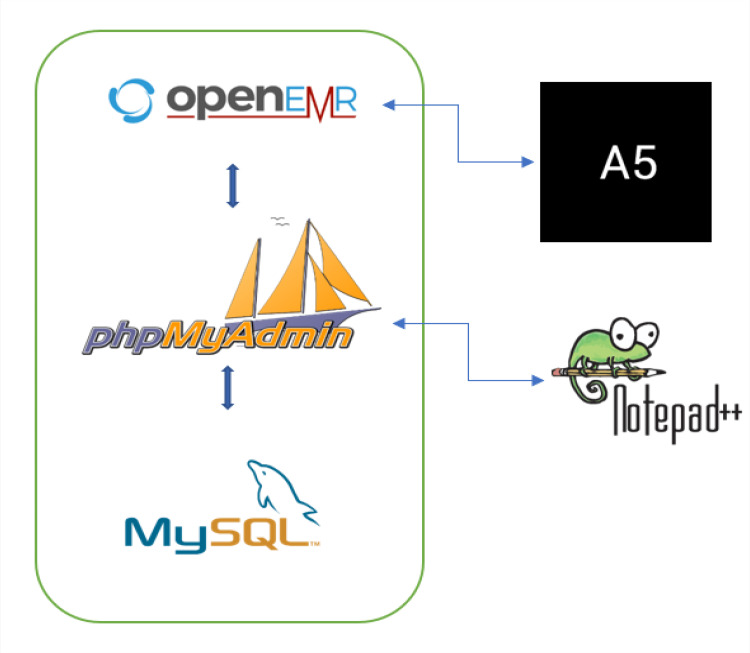
Selection flow chart of development tools.

Consequently, we came to use the PrintPatient_A5 tool for better compliance with OpenEMR. PrintPatient_A5 is a specialized patient data generator adapted to meet the requirements of OpenEMR. It is necessary to describe the diseases, the causes, the symptoms, and the ranges of normal values. Table comparison in the database, after each update or insertion, is conducted with Notepad++. The following figure illustrates the layout of the software components which compose our proposal (Figure 2).

## Results

Software overview

OpenEMR

**Figure 3:**
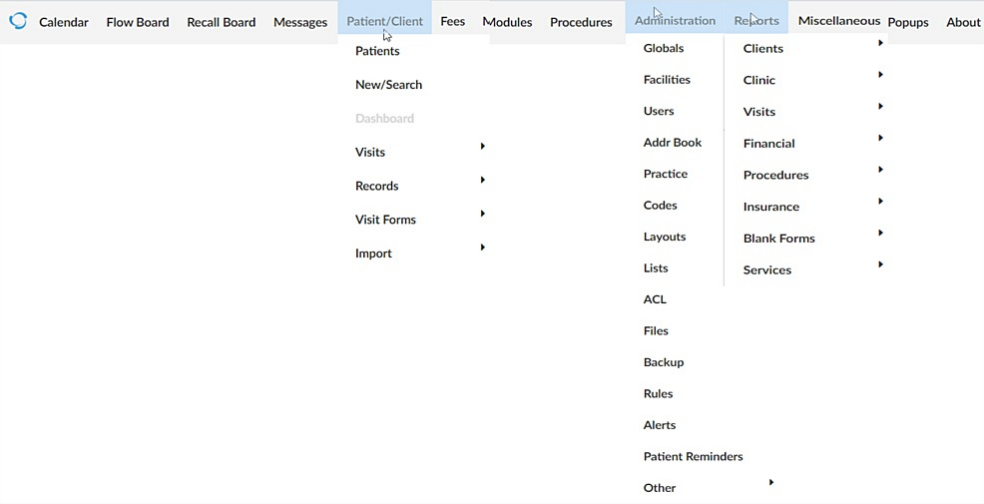
The OpenEMR main menu.

As discussed before, the solution presented in this study is based on OpenEMR. In Figure 3, the options offered by OpenEMR in the main menu are presented. Then, indicatively, we look up the options offered by some of the menus. In particular, we have expanded the "Patient/Client" option, the "Administration" option, and the "Reports" option. Apparently, OpenEMR offers a superset of capabilities needed for the design of the prototype. Therefore, we need to determine the necessary capabilities for the design of the prototype.

phpMyAdmin

**Figure 4:**
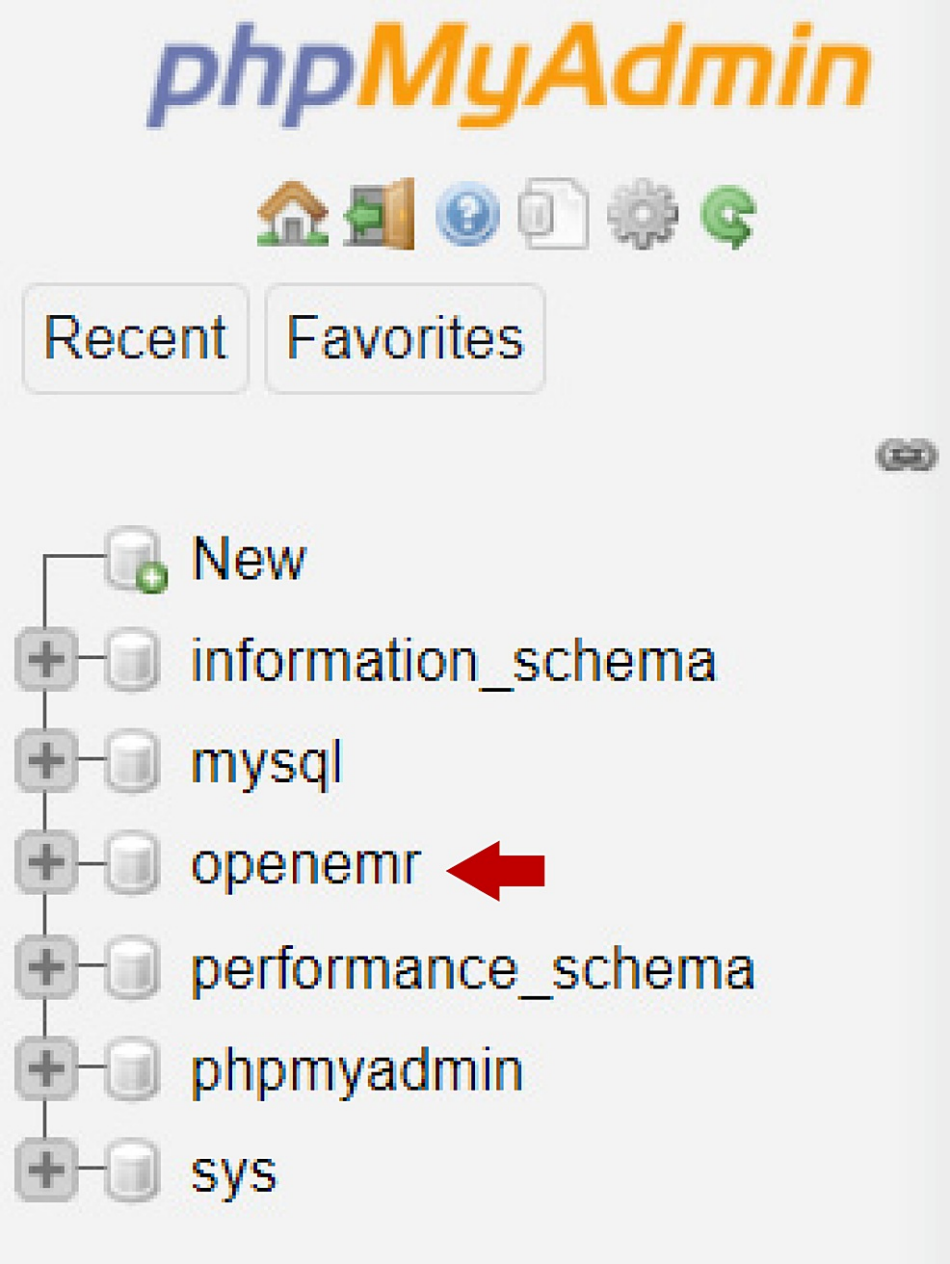
The phpMyAdmin installation.

The phpMyAdmin application was installed and commissioned along with the OpenEMR installation. phpMyAdmin is a free web-based administration tool for MySQL, which is the database of OpenEMR. According to the following list (Figure 4), the name of the database is openemr.

Data and records

Data Entry

As the next steps, we have populated the disease coding data, the patient demographics, and blood test reference values (n=15). Again, data entry was performed via OpenEMR.

Import and Update of ICD10

The International Classification of Diseases (ICD) nomenclature is a medical classification list introduced by the WHO. It contains coded information about diseases, symptoms, diagnoses, pathological findings, and generally anything that may be directly related to a disease. ICD-10 is the tenth revision, and its latest version is of 2019. ICD-10 update is performed via OpenEMR. From the main menu, the selection process was "Administration" → "Other" → "External Data Loads." Then, under "External Database Import Utility," we chose the ICD-10 list. Thus, we updated the OpenEMR with the 2019 version of ICD-10.

Dummy Patients Utility

For the purpose of creating a dummy patient cohort, we used the PrintPatient_A5 tool, a specialized patient data generator adapted to meet the requirements of OpenEMR. PrintPatient_A5 creates patients' demographics, which match precisely into the appropriate fields of the patient cards.

Comparison of Tables

Table comparison in the database, after each update or insertion, is conducted with Notepad++. Notepad++ is an open-source text editor with a plethora of programming tools. With this editor, we compared snapshots of the same database before and after insertion or update. In that way, we could identify which tables were modified.

Creating Records and Analyzing the Database

The purpose of the following task was to identify the updated tables after entering or modifying data in OpenEMR. The scenario concerns patients with thyroid disease. For simplicity, the scenario concerned adult patients. That way, we avoided the complexity of sub-cases due to factors such as the patient's age and sex. It is emphasized that data and information regarding dummy patients and the outcomes are false. The objective is to test the capabilities of OpenEMR for the design of the patient recruitment application, not the accuracy of the values and results.

A series of steps need to be taken to update the EHRs of dummy patients with laboratory test results. The objective of these steps was to identify which database tables are affected when updating OpenEMR. For example, these tables may be the infrastructure that may support the design of patient recruitment applications. The work steps follow the instructions on the official OpenEMR website regarding the manual entry of lab tests and results.

Initially, a "clean" database was created by installing OpenEMR and was subsequently updated with ICD-10 encoding. Icd10_dx_order_code is the modified table. As the next step, the dummy Endocrinology Lab is inserted. The laboratory is necessary for ordering laboratory tests, and users and procedure_providers tables are updated. Then, appropriate laboratory tests for hypothyroidism, hyperparathyroidism, and nodular goiter are inserted, and list_options and procedure_type tables are updated.

Further on, OpenEMR is updated by importing patient dummy tabs, and the patient_data table is updated. Then, patients' records are updated with disease imports. The imports are conducted by ICD-10 encoding, and lists and lists_touch tables are updated. The first table is updated with the medical problem for each patient, while the second table is updated with disease admissions for each patient. Thereafter, patients are informed of medical meetings necessary to place the procedure orders. The updated tables are forms and form_encounter. Then, the procedure orders are entered. The updated tables are: a) procedure_order, b) procedure_order_code, and c) procedure_type. Finally, the EHRs of patients are updated with the results of laboratory tests. The updated tables are procedure_report and procedure_result.

**Figure 5:**
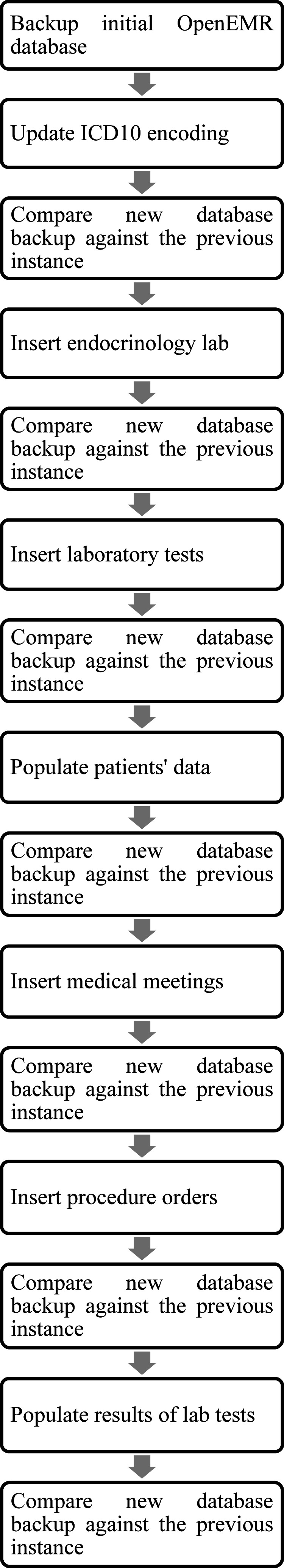
Flowchart of steps of OpenEMR-derived updated tables.

The following flowchart describes the appropriate steps of OpenEMR-derived updated tables (Figure 5).

In summary, the tables affected by the updates are: icd10_dx_order_code, users, procedure_providers, list_options, procedure_type, patient_data, lists, lists_touch, forms, form_encounter, procedure_order, procedure_order_code, procedure_type, procedure_report, and procedure_result. The update of the procedure_type table takes place twice. Thus, the number of updated tables is fourteen. Below are the structures of the aforementioned tables shown in Table 3. Code was obtained and adapted from OpenEMR [20] under the GNU General Public License (GNU GPL).

**Table 3:** The structures of the database tables. Code obtained and adapted from OpenEMR [20].

icd10_dx_order_code
“CREATE TABLE `icd10_dx_order_code` ( `dx_id` bigint(20) UNSIGNED NOT NULL, `dx_code` varchar(7) DEFAULT NULL, `formatted_dx_code` varchar(10) DEFAULT NULL, `valid_for_coding` char(1) DEFAULT NULL, `short_desc` varchar(60) DEFAULT NULL, `long_desc` varchar(300) DEFAULT NULL, `active` tinyint(4) DEFAULT '0', `revision` int(11) DEFAULT '0' ) ENGINE=InnoDB DEFAULT CHARSET=utf8;”
Users
“CREATE TABLE `users` ( `id` bigint(20) NOT NULL, `username` varchar(255) DEFAULT NULL, `password` longtext, `authorized` tinyint(4) DEFAULT NULL, `info` longtext, `source` tinyint(4) DEFAULT NULL, `fname` varchar(255) DEFAULT NULL, `mname` varchar(255) DEFAULT NULL, `lname` varchar(255) DEFAULT NULL, `suffix` varchar(255) DEFAULT NULL, `federaltaxid` varchar(255) DEFAULT NULL, `federaldrugid` varchar(255) DEFAULT NULL, `upin` varchar(255) DEFAULT NULL, `facility` varchar(255) DEFAULT NULL, `facility_id` int(11) NOT NULL DEFAULT '0', `see_auth` int(11) NOT NULL DEFAULT '1', `active` tinyint(1) NOT NULL DEFAULT '1', `npi` varchar(15) DEFAULT NULL, `title` varchar(30) DEFAULT NULL, `specialty` varchar(255) DEFAULT NULL, `billname` varchar(255) DEFAULT NULL, `email` varchar(255) DEFAULT NULL, `email_direct` varchar(255) NOT NULL DEFAULT '', `url` varchar(255) DEFAULT NULL, `assistant` varchar(255) DEFAULT NULL, `organization` varchar(255) DEFAULT NULL, `valedictory` varchar(255) DEFAULT NULL, `street` varchar(60) DEFAULT NULL, `streetb` varchar(60) DEFAULT NULL, `city` varchar(30) DEFAULT NULL, `state` varchar(30) DEFAULT NULL, `zip` varchar(20) DEFAULT NULL, `street2` varchar(60) DEFAULT NULL, `streetb2` varchar(60) DEFAULT NULL, `city2` varchar(30) DEFAULT NULL, `state2` varchar(30) DEFAULT NULL, `zip2` varchar(20) DEFAULT NULL, `phone` varchar(30) DEFAULT NULL, `fax` varchar(30) DEFAULT NULL, `phonew1` varchar(30) DEFAULT NULL, `phonew2` varchar(30) DEFAULT NULL, `phonecell` varchar(30) DEFAULT NULL, `notes` text, `cal_ui` tinyint(4) NOT NULL DEFAULT '1', `taxonomy` varchar(30) NOT NULL DEFAULT '207Q00000X', `calendar` tinyint(1) NOT NULL DEFAULT '0' COMMENT '1 = appears in calendar', `abook_type` varchar(31) NOT NULL DEFAULT '', `pwd_expiration_date` date DEFAULT NULL, `pwd_history1` longtext, `pwd_history2` longtext, `default_warehouse` varchar(31) NOT NULL DEFAULT '', `irnpool` varchar(31) NOT NULL DEFAULT '', `state_license_number` varchar(25) DEFAULT NULL, `weno_prov_id` varchar(15) DEFAULT NULL, `newcrop_user_role` varchar(30) DEFAULT NULL, `cpoe` tinyint(1) DEFAULT NULL, `physician_type` varchar(50) DEFAULT NULL, `main_menu_role` varchar(50) NOT NULL DEFAULT 'standard', `patient_menu_role` varchar(50) NOT NULL DEFAULT 'standard' ) ENGINE=InnoDB DEFAULT CHARSET=latin1;”
procedure_providers
“CREATE TABLE `procedure_providers` ( `ppid` bigint(20) NOT NULL, `name` varchar(255) NOT NULL DEFAULT '', `npi` varchar(15) NOT NULL DEFAULT '', `send_app_id` varchar(255) NOT NULL DEFAULT '' COMMENT 'Sending application ID (MSH-3.1)', `send_fac_id` varchar(255) NOT NULL DEFAULT '' COMMENT 'Sending facility ID (MSH-4.1)', `recv_app_id` varchar(255) NOT NULL DEFAULT '' COMMENT 'Receiving application ID (MSH-5.1)', `recv_fac_id` varchar(255) NOT NULL DEFAULT '' COMMENT 'Receiving facility ID (MSH-6.1)', `DorP` char(1) NOT NULL DEFAULT 'D' COMMENT 'Debugging or Production (MSH-11)', `direction` char(1) NOT NULL DEFAULT 'B' COMMENT 'Bidirectional or Results-only', `protocol` varchar(15) NOT NULL DEFAULT 'DL', `remote_host` varchar(255) NOT NULL DEFAULT '', `login` varchar(255) NOT NULL DEFAULT '', `password` varchar(255) NOT NULL DEFAULT '', `orders_path` varchar(255) NOT NULL DEFAULT '', `results_path` varchar(255) NOT NULL DEFAULT '', `notes` text, `lab_director` bigint(20) NOT NULL DEFAULT '0' ) ENGINE=InnoDB DEFAULT CHARSET=latin1;”
list_options
“CREATE TABLE `list_options` ( `list_id` varchar(100) NOT NULL DEFAULT '', `option_id` varchar(100) NOT NULL DEFAULT '', `title` varchar(255) NOT NULL DEFAULT '', `seq` int(11) NOT NULL DEFAULT '0', `is_default` tinyint(1) NOT NULL DEFAULT '0', `option_value` float NOT NULL DEFAULT '0', `mapping` varchar(31) NOT NULL DEFAULT '', `notes` text, `codes` varchar(255) NOT NULL DEFAULT '', `toggle_setting_1` tinyint(1) NOT NULL DEFAULT '0', `toggle_setting_2` tinyint(1) NOT NULL DEFAULT '0', `activity` tinyint(4) NOT NULL DEFAULT '1', `subtype` varchar(31) NOT NULL DEFAULT '', `edit_options` tinyint(1) NOT NULL DEFAULT '1', `timestamp` timestamp NOT NULL DEFAULT CURRENT_TIMESTAMP ) ENGINE=InnoDB DEFAULT CHARSET=latin1;”
procedure_type
“CREATE TABLE `procedure_type` ( `procedure_type_id` bigint(20) NOT NULL, `parent` bigint(20) NOT NULL DEFAULT '0' COMMENT 'references procedure_type.procedure_type_id', `name` varchar(63) NOT NULL DEFAULT '' COMMENT 'name for this category, procedure or result type', `lab_id` bigint(20) NOT NULL DEFAULT '0' COMMENT 'references procedure_providers.ppid, 0 means default to parent', `procedure_code` varchar(31) NOT NULL DEFAULT '' COMMENT 'code identifying this procedure', `procedure_type` varchar(31) NOT NULL DEFAULT '' COMMENT 'see list proc_type', `body_site` varchar(31) NOT NULL DEFAULT '' COMMENT 'where to do injection, e.g. arm, buttock', `specimen` varchar(31) NOT NULL DEFAULT '' COMMENT 'blood, urine, saliva, etc.', `route_admin` varchar(31) NOT NULL DEFAULT '' COMMENT 'oral, injection', `laterality` varchar(31) NOT NULL DEFAULT '' COMMENT 'left, right, ...', `description` varchar(255) NOT NULL DEFAULT '' COMMENT 'descriptive text for procedure_code', `standard_code` varchar(255) NOT NULL DEFAULT '' COMMENT 'industry standard code type and code (e.g. CPT4:12345)', `related_code` varchar(255) NOT NULL DEFAULT '' COMMENT 'suggested code(s) for followup services if result is abnormal', `units` varchar(31) NOT NULL DEFAULT '' COMMENT 'default for procedure_result.units', `range` varchar(255) NOT NULL DEFAULT '' COMMENT 'default for procedure_result.range', `seq` int(11) NOT NULL DEFAULT '0' COMMENT 'sequence number for ordering', `activity` tinyint(1) NOT NULL DEFAULT '1' COMMENT '1=active, 0=inactive', `notes` varchar(255) NOT NULL DEFAULT '' COMMENT 'additional notes to enhance description' ) ENGINE=InnoDB DEFAULT CHARSET=latin1;”
patient_data
“CREATE TABLE `patient_data` ( `id` bigint(20) NOT NULL, `title` varchar(255) NOT NULL DEFAULT '', `language` varchar(255) NOT NULL DEFAULT '', `financial` varchar(255) NOT NULL DEFAULT '', `fname` varchar(255) NOT NULL DEFAULT '', `lname` varchar(255) NOT NULL DEFAULT '', `mname` varchar(255) NOT NULL DEFAULT '', `DOB` date DEFAULT NULL, `street` varchar(255) NOT NULL DEFAULT '', `postal_code` varchar(255) NOT NULL DEFAULT '', `city` varchar(255) NOT NULL DEFAULT '', `state` varchar(255) NOT NULL DEFAULT '', `country_code` varchar(255) NOT NULL DEFAULT '', `drivers_license` varchar(255) NOT NULL DEFAULT '', `ss` varchar(255) NOT NULL DEFAULT '', `occupation` longtext, `phone_home` varchar(255) NOT NULL DEFAULT '', `phone_biz` varchar(255) NOT NULL DEFAULT '', `phone_contact` varchar(255) NOT NULL DEFAULT '', `phone_cell` varchar(255) NOT NULL DEFAULT '', `pharmacy_id` int(11) NOT NULL DEFAULT '0', `status` varchar(255) NOT NULL DEFAULT '', `contact_relationship` varchar(255) NOT NULL DEFAULT '', `date` datetime DEFAULT NULL, `sex` varchar(255) NOT NULL DEFAULT '', `referrer` varchar(255) NOT NULL DEFAULT '', `referrerID` varchar(255) NOT NULL DEFAULT '', `providerID` int(11) DEFAULT NULL, `ref_providerID` int(11) DEFAULT NULL, `email` varchar(255) NOT NULL DEFAULT '', `email_direct` varchar(255) NOT NULL DEFAULT '', `ethnoracial` varchar(255) NOT NULL DEFAULT '', `race` varchar(255) NOT NULL DEFAULT '', `ethnicity` varchar(255) NOT NULL DEFAULT '', `religion` varchar(40) NOT NULL DEFAULT '', `interpretter` varchar(255) NOT NULL DEFAULT '', `migrantseasonal` varchar(255) NOT NULL DEFAULT '', `family_size` varchar(255) NOT NULL DEFAULT '', `monthly_income` varchar(255) NOT NULL DEFAULT '', `billing_note` text, `homeless` varchar(255) NOT NULL DEFAULT '', `financial_review` datetime DEFAULT NULL, `pubpid` varchar(255) NOT NULL DEFAULT '', `pid` bigint(20) NOT NULL DEFAULT '0', `genericname1` varchar(255) NOT NULL DEFAULT '', `genericval1` varchar(255) NOT NULL DEFAULT '', `genericname2` varchar(255) NOT NULL DEFAULT '', `genericval2` varchar(255) NOT NULL DEFAULT '', `hipaa_mail` varchar(3) NOT NULL DEFAULT '', `hipaa_voice` varchar(3) NOT NULL DEFAULT '', `hipaa_notice` varchar(3) NOT NULL DEFAULT '', `hipaa_message` varchar(20) NOT NULL DEFAULT '', `hipaa_allowsms` varchar(3) NOT NULL DEFAULT 'NO', `hipaa_allowemail` varchar(3) NOT NULL DEFAULT 'NO', `squad` varchar(32) NOT NULL DEFAULT '', `fitness` int(11) NOT NULL DEFAULT '0', `referral_source` varchar(30) NOT NULL DEFAULT '', `usertext1` varchar(255) NOT NULL DEFAULT '', `usertext2` varchar(255) NOT NULL DEFAULT '', `usertext3` varchar(255) NOT NULL DEFAULT '', `usertext4` varchar(255) NOT NULL DEFAULT '', `usertext5` varchar(255) NOT NULL DEFAULT '', `usertext6` varchar(255) NOT NULL DEFAULT '', `usertext7` varchar(255) NOT NULL DEFAULT '', `usertext8` varchar(255) NOT NULL DEFAULT '', `userlist1` varchar(255) NOT NULL DEFAULT '', `userlist2` varchar(255) NOT NULL DEFAULT '', `userlist3` varchar(255) NOT NULL DEFAULT '', `userlist4` varchar(255) NOT NULL DEFAULT '', `userlist5` varchar(255) NOT NULL DEFAULT '', `userlist6` varchar(255) NOT NULL DEFAULT '', `userlist7` varchar(255) NOT NULL DEFAULT '', `pricelevel` varchar(255) NOT NULL DEFAULT 'standard', `regdate` date DEFAULT NULL COMMENT 'Registration Date', `contrastart` date DEFAULT NULL COMMENT 'Date contraceptives initially used', `completed_ad` varchar(3) NOT NULL DEFAULT 'NO', `ad_reviewed` date DEFAULT NULL, `vfc` varchar(255) NOT NULL DEFAULT '', `mothersname` varchar(255) NOT NULL DEFAULT '', `guardiansname` text, `allow_imm_reg_use` varchar(255) NOT NULL DEFAULT '', `allow_imm_info_share` varchar(255) NOT NULL DEFAULT '', `allow_health_info_ex` varchar(255) NOT NULL DEFAULT '', `allow_patient_portal` varchar(31) NOT NULL DEFAULT '', `deceased_date` datetime DEFAULT NULL, `deceased_reason` varchar(255) NOT NULL DEFAULT '', `soap_import_status` tinyint(4) DEFAULT NULL COMMENT '1-Prescription Press 2-Prescription Import 3-Allergy Press 4-Allergy Import', `cmsportal_login` varchar(60) NOT NULL DEFAULT '', `care_team` int(11) DEFAULT NULL, `county` varchar(40) NOT NULL DEFAULT '', `industry` text, `imm_reg_status` text, `imm_reg_stat_effdate` text, `publicity_code` text, `publ_code_eff_date` text, `protect_indicator` text, `prot_indi_effdate` text, `guardianrelationship` text, `guardiansex` text, `guardianaddress` text, `guardiancity` text, `guardianstate` text, `guardianpostalcode` text, `guardiancountry` text, `guardianphone` text, `guardianworkphone` text, `guardianemail` text ) ENGINE=InnoDB DEFAULT CHARSET=latin1;”
Lists
“CREATE TABLE `lists` ( `id` bigint(20) NOT NULL, `date` datetime DEFAULT NULL, `type` varchar(255) DEFAULT NULL, `subtype` varchar(31) NOT NULL DEFAULT '', `title` varchar(255) DEFAULT NULL, `begdate` date DEFAULT NULL, `enddate` date DEFAULT NULL, `returndate` date DEFAULT NULL, `occurrence` int(11) DEFAULT '0', `classification` int(11) DEFAULT '0', `referredby` varchar(255) DEFAULT NULL, `extrainfo` varchar(255) DEFAULT NULL, `diagnosis` varchar(255) DEFAULT NULL, `activity` tinyint(4) DEFAULT NULL, `comments` longtext, `pid` bigint(20) DEFAULT NULL, `user` varchar(255) DEFAULT NULL, `groupname` varchar(255) DEFAULT NULL, `outcome` int(11) NOT NULL DEFAULT '0', `destination` varchar(255) DEFAULT NULL, `reinjury_id` bigint(20) NOT NULL DEFAULT '0', `injury_part` varchar(31) NOT NULL DEFAULT '', `injury_type` varchar(31) NOT NULL DEFAULT '', `injury_grade` varchar(31) NOT NULL DEFAULT '', `reaction` varchar(255) NOT NULL DEFAULT '', `external_allergyid` int(11) DEFAULT NULL, `erx_source` enum('0','1') NOT NULL DEFAULT '0' COMMENT '0-OpenEMR 1-External', `erx_uploaded` enum('0','1') NOT NULL DEFAULT '0' COMMENT '0-Pending NewCrop upload 1-Uploaded TO NewCrop', `modifydate` timestamp NOT NULL DEFAULT CURRENT_TIMESTAMP ON UPDATE CURRENT_TIMESTAMP, `severity_al` varchar(50) DEFAULT NULL, `external_id` varchar(20) DEFAULT NULL, `list_option_id` varchar(100) DEFAULT NULL COMMENT 'Reference to list_options table' ) ENGINE=InnoDB DEFAULT CHARSET=latin1;”
lists_touch
“CREATE TABLE `lists_touch` ( `pid` bigint(20) NOT NULL DEFAULT '0', `type` varchar(255) NOT NULL DEFAULT '', `date` datetime DEFAULT NULL ) ENGINE=InnoDB DEFAULT CHARSET=latin1;”
Forms
“CREATE TABLE `forms` ( `id` bigint(20) NOT NULL, `date` datetime DEFAULT NULL, `encounter` bigint(20) DEFAULT NULL, `form_name` longtext, `form_id` bigint(20) DEFAULT NULL, `pid` bigint(20) DEFAULT NULL, `user` varchar(255) DEFAULT NULL, `groupname` varchar(255) DEFAULT NULL, `authorized` tinyint(4) DEFAULT NULL, `deleted` tinyint(4) NOT NULL DEFAULT '0' COMMENT 'flag indicates form has been deleted', `formdir` longtext, `therapy_group_id` int(11) DEFAULT NULL, `issue_id` bigint(20) NOT NULL DEFAULT '0' COMMENT 'references lists.id to identify a case', `provider_id` bigint(20) NOT NULL DEFAULT '0' COMMENT 'references users.id to identify a provider' ) ENGINE=InnoDB DEFAULT CHARSET=latin1;”
form_encounter
“CREATE TABLE `form_encounter` ( `id` bigint(20) NOT NULL, `date` datetime DEFAULT NULL, `reason` longtext, `facility` longtext, `facility_id` int(11) NOT NULL DEFAULT '0', `pid` bigint(20) DEFAULT NULL, `encounter` bigint(20) DEFAULT NULL, `onset_date` datetime DEFAULT NULL, `sensitivity` varchar(30) DEFAULT NULL, `billing_note` text, `pc_catid` int(11) NOT NULL DEFAULT '5' COMMENT 'event category from openemr_postcalendar_categories', `last_level_billed` int(11) NOT NULL DEFAULT '0' COMMENT '0=none, 1=ins1, 2=ins2, etc', `last_level_closed` int(11) NOT NULL DEFAULT '0' COMMENT '0=none, 1=ins1, 2=ins2, etc', `last_stmt_date` date DEFAULT NULL, `stmt_count` int(11) NOT NULL DEFAULT '0', `provider_id` int(11) DEFAULT '0' COMMENT 'default and main provider for this visit', `supervisor_id` int(11) DEFAULT '0' COMMENT 'supervising provider, if any, for this visit', `invoice_refno` varchar(31) NOT NULL DEFAULT '', `referral_source` varchar(31) NOT NULL DEFAULT '', `billing_facility` int(11) NOT NULL DEFAULT '0', `external_id` varchar(20) DEFAULT NULL, `pos_code` tinyint(4) DEFAULT NULL ) ENGINE=InnoDB DEFAULT CHARSET=latin1;”
procedure_order
“CREATE TABLE `procedure_order` ( `procedure_order_id` bigint(20) NOT NULL, `provider_id` bigint(20) NOT NULL DEFAULT '0' COMMENT 'references users.id, the ordering provider', `patient_id` bigint(20) NOT NULL COMMENT 'references patient_data.pid', `encounter_id` bigint(20) NOT NULL DEFAULT '0' COMMENT 'references form_encounter.encounter', `date_collected` datetime DEFAULT NULL COMMENT 'time specimen collected', `date_ordered` date DEFAULT NULL, `order_priority` varchar(31) NOT NULL DEFAULT '', `order_status` varchar(31) NOT NULL DEFAULT '' COMMENT 'pending,routed,complete,canceled', `patient_instructions` text, `activity` tinyint(1) NOT NULL DEFAULT '1' COMMENT '0 if deleted', `control_id` varchar(255) NOT NULL DEFAULT '' COMMENT 'This is the CONTROL ID that is sent back from lab', `lab_id` bigint(20) NOT NULL DEFAULT '0' COMMENT 'references procedure_providers.ppid', `specimen_type` varchar(31) NOT NULL DEFAULT '' COMMENT 'from the Specimen_Type list', `specimen_location` varchar(31) NOT NULL DEFAULT '' COMMENT 'from the Specimen_Location list', `specimen_volume` varchar(30) NOT NULL DEFAULT '' COMMENT 'from a text input field', `date_transmitted` datetime DEFAULT NULL COMMENT 'time of order transmission, null if unsent', `clinical_hx` varchar(255) NOT NULL DEFAULT '' COMMENT 'clinical history text that may be relevant to the order', `external_id` varchar(20) DEFAULT NULL, `history_order` enum('0','1') DEFAULT '0' COMMENT 'references order is added for history purpose only.', `order_diagnosis` varchar(255) DEFAULT '' COMMENT 'primary order diagnosis' ) ENGINE=InnoDB DEFAULT CHARSET=latin1;”
procedure_order_code
“CREATE TABLE `procedure_order_code` ( `procedure_order_id` bigint(20) NOT NULL COMMENT 'references procedure_order.procedure_order_id', `procedure_order_seq` int(11) NOT NULL COMMENT 'Supports multiple tests per order. Procedure_order_seq, incremented in code', `procedure_code` varchar(31) NOT NULL DEFAULT '' COMMENT 'like procedure_type.procedure_code', `procedure_name` varchar(255) NOT NULL DEFAULT '' COMMENT 'descriptive name of the procedure code', `procedure_source` char(1) NOT NULL DEFAULT '1' COMMENT '1=original order, 2=added after order sent', `diagnoses` text COMMENT 'diagnoses and maybe other coding (e.g. ICD9:111.11)', `do_not_send` tinyint(1) NOT NULL DEFAULT '0' COMMENT '0 = normal, 1 = do not transmit to lab', `procedure_order_title` varchar(255) DEFAULT NULL ) ENGINE=InnoDB DEFAULT CHARSET=latin1;”
procedure_report
“CREATE TABLE `procedure_report` ( `procedure_report_id` bigint(20) NOT NULL, `procedure_order_id` bigint(20) DEFAULT NULL COMMENT 'references procedure_order.procedure_order_id', `procedure_order_seq` int(11) NOT NULL DEFAULT '1' COMMENT 'references procedure_order_code.procedure_order_seq', `date_collected` datetime DEFAULT NULL, `date_collected_tz` varchar(5) DEFAULT '' COMMENT '+-hhmm offset from UTC', `date_report` datetime DEFAULT NULL, `date_report_tz` varchar(5) DEFAULT '' COMMENT '+-hhmm offset from UTC', `source` bigint(20) NOT NULL DEFAULT '0' COMMENT 'references users.id, who entered this data', `specimen_num` varchar(63) NOT NULL DEFAULT '', `report_status` varchar(31) NOT NULL DEFAULT '' COMMENT 'received,complete,error', `review_status` varchar(31) NOT NULL DEFAULT 'received' COMMENT 'pending review status: received,reviewed', `report_notes` text COMMENT 'notes from the lab' ) ENGINE=InnoDB DEFAULT CHARSET=latin1;”
procedure_result
“CREATE TABLE `procedure_result` ( `procedure_result_id` bigint(20) NOT NULL, `procedure_report_id` bigint(20) NOT NULL COMMENT 'references procedure_report.procedure_report_id', `result_data_type` char(1) NOT NULL DEFAULT 'S' COMMENT 'N=Numeric, S=String, F=Formatted, E=External, L=Long text as first line of comments', `result_code` varchar(31) NOT NULL DEFAULT '' COMMENT 'LOINC code, might match a procedure_type.procedure_code', `result_text` varchar(255) NOT NULL DEFAULT '' COMMENT 'Description of result_code', `date` datetime DEFAULT NULL COMMENT 'lab-provided date specific to this result', `facility` varchar(255) NOT NULL DEFAULT '' COMMENT 'lab-provided testing facility ID', `units` varchar(31) NOT NULL DEFAULT '', `result` varchar(255) NOT NULL DEFAULT '', `range` varchar(255) NOT NULL DEFAULT '', `abnormal` varchar(31) NOT NULL DEFAULT '' COMMENT 'no,yes,high,low', `comments` text COMMENT 'comments from the lab', `document_id` bigint(20) NOT NULL DEFAULT '0' COMMENT 'references documents.id if this result is a document', `result_status` varchar(31) NOT NULL DEFAULT '' COMMENT 'preliminary, cannot be done, final, corrected, incomplete...etc.' ) ENGINE=InnoDB DEFAULT CHARSET=latin1;”

Once the affected tables by the OpenEMR update are identified, it is necessary to check which tables have the appropriate data to design the prototype. Indicatively, we state that necessary data are the patients' demographics, the diseases, the range of normal values, and test results. Hence, it is necessary to examine the structure and fields of the tables so we can proceed to the final selection. For the present study, it was necessary to decide the minimum of information that satisfies the requirements of the patient recruitment prototype. A minimum of information may contain the following fields: a) demographics, b) laboratory tests, c) procedure results, d) units of measurement, e) reference ranges, and f) abnormal values. Thus, the tables may be modified as follows as in Table 4. Code was obtained and adapted from OpenEMR [20] under the GNU General Public License (GNU GPL).

**Table 4:** The structures of the database tables containing the minimum information containing the following fields: : a) demographics, b) laboratory tests, c) procedure results, d) units of measurement, e) reference ranges, and f) abnormal values. Code was obtained and adapted from OpenEMR [20].

icd10_dx_order_code (ICD-10 ENCODING)
“CREATE TABLE `icd10_dx_order_code` ( `dx_id` bigint(20) UNSIGNED NOT NULL, `formatted_dx_code` varchar(10) DEFAULT NULL, `short_desc` varchar(60) DEFAULT NULL ) ENGINE=InnoDB DEFAULT CHARSET=utf8;”
list_options (LABORATORY MEASUREMENT UNITS)
“CREATE TABLE `list_options` ( `list_id` varchar(100) NOT NULL DEFAULT '', `option_id` varchar(100) NOT NULL DEFAULT '', `title` varchar(255) NOT NULL DEFAULT '', `seq` int(11) NOT NULL DEFAULT '0', `is_default` tinyint(1) NOT NULL DEFAULT '0', `option_value` float NOT NULL DEFAULT '0', `mapping` varchar(31) NOT NULL DEFAULT '', `notes` text ) ENGINE=InnoDB DEFAULT CHARSET=latin1;”
procedure_type (PROCEDURES)
“CREATE TABLE `procedure_type` ( `procedure_type_id` bigint(20) NOT NULL, `parent` bigint(20) NOT NULL DEFAULT '0' COMMENT 'references procedure_type.procedure_type_id', `name` varchar(63) NOT NULL DEFAULT '' COMMENT 'name for this category, procedure or result type', `procedure_code` varchar(31) NOT NULL DEFAULT '' COMMENT 'code identifying this procedure', `procedure_type` varchar(31) NOT NULL DEFAULT '' COMMENT 'see list proc_type', `description` varchar(255) NOT NULL DEFAULT '' COMMENT 'descriptive text for procedure_code', `units` varchar(31) NOT NULL DEFAULT '' COMMENT 'default for procedure_result.units', `range` varchar(255) NOT NULL DEFAULT '' COMMENT 'default for procedure_result.range', `seq` int(11) NOT NULL DEFAULT '0' COMMENT 'sequence number for ordering', `activity` tinyint(1) NOT NULL DEFAULT '1' COMMENT '1=active, 0=inactive' ) ENGINE=InnoDB DEFAULT CHARSET=latin1;”
patient_data (PATIENTS’ TABLE)
“CREATE TABLE `patient_data` ( `id` bigint(20) NOT NULL, `title` varchar(255) NOT NULL DEFAULT '', `fname` varchar(255) NOT NULL DEFAULT '', `lname` varchar(255) NOT NULL DEFAULT '', `mname` varchar(255) NOT NULL DEFAULT '', `DOB` date DEFAULT NULL, `ss` varchar(255) NOT NULL DEFAULT '', `phone_cell` varchar(255) NOT NULL DEFAULT '', `email` varchar(255) NOT NULL DEFAULT '', `pid` bigint(20) NOT NULL DEFAULT '0' ) ENGINE=InnoDB DEFAULT CHARSET=latin1;”
Lists (DISEASES PER PATIENT)
“CREATE TABLE `lists` ( `id` bigint(20) NOT NULL, `type` varchar(255) DEFAULT NULL, `title` varchar(255) DEFAULT NULL, `diagnosis` varchar(255) DEFAULT NULL, `pid` bigint(20) DEFAULT NULL ) ENGINE=InnoDB DEFAULT CHARSET=latin1;”
forms (MEDICAL SESSIONS)
“CREATE TABLE `forms` ( `id` bigint(20) NOT NULL, `encounter` bigint(20) DEFAULT NULL, `form_name` longtext, `form_id` bigint(20) DEFAULT NULL, `pid` bigint(20) DEFAULT NULL ) ENGINE=InnoDB DEFAULT CHARSET=latin1;”
form_encounter (MEDICAL SESSIONS PER PATIENT)
“CREATE TABLE `form_encounter` ( `id` bigint(20) NOT NULL, `date` datetime DEFAULT NULL, `pid` bigint(20) DEFAULT NULL, `encounter` bigint(20) DEFAULT NULL ) ENGINE=InnoDB DEFAULT CHARSET=latin1;”
procedure_order
“CREATE TABLE `procedure_order` ( `procedure_order_id` bigint(20) NOT NULL, `provider_id` bigint(20) NOT NULL DEFAULT '0' COMMENT 'references users.id, the ordering provider', `patient_id` bigint(20) NOT NULL COMMENT 'references patient_data.pid', `date_ordered` date DEFAULT NULL ) ENGINE=InnoDB DEFAULT CHARSET=latin1;”
procedure_order_code
“CREATE TABLE `procedure_order_code` ( `procedure_order_id` bigint(20) NOT NULL COMMENT 'references procedure_order.procedure_order_id', `procedure_order_seq` int(11) NOT NULL COMMENT 'Supports multiple tests per order. Procedure_order_seq, incremented in code', `procedure_code` varchar(31) NOT NULL DEFAULT '' COMMENT 'like procedure_type.procedure_code', `procedure_name` varchar(255) NOT NULL DEFAULT '' COMMENT 'descriptive name of the procedure code' ) ENGINE=InnoDB DEFAULT CHARSET=latin1;”
procedure_report
“CREATE TABLE `procedure_report` ( `procedure_report_id` bigint(20) NOT NULL, `procedure_order_id` bigint(20) DEFAULT NULL COMMENT 'references procedure_order.procedure_order_id', `procedure_order_seq` int(11) NOT NULL DEFAULT '1' COMMENT 'references procedure_order_code.procedure_order_seq', `specimen_num` varchar(63) NOT NULL DEFAULT '' ) ENGINE=InnoDB DEFAULT CHARSET=latin1;”
procedure_result
“CREATE TABLE `procedure_result` ( `procedure_result_id` bigint(20) NOT NULL, `procedure_report_id` bigint(20) NOT NULL COMMENT 'references procedure_report.procedure_report_id', `result_data_type` char(1) NOT NULL DEFAULT 'S' COMMENT 'N=Numeric, S=String, F=Formatted, E=External, L=Long text as first line of comments', `result_code` varchar(31) NOT NULL DEFAULT '' COMMENT 'LOINC code, might match a procedure_type.procedure_code', `result_text` varchar(255) NOT NULL DEFAULT '' COMMENT 'Description of result_code', `date` datetime DEFAULT NULL COMMENT 'lab-provided date specific to this result', `facility` varchar(255) NOT NULL DEFAULT '' COMMENT 'lab-provided testing facility ID', `units` varchar(31) NOT NULL DEFAULT '', `result` varchar(255) NOT NULL DEFAULT '', `range` varchar(255) NOT NULL DEFAULT '', `abnormal` varchar(31) NOT NULL DEFAULT '' COMMENT 'no,yes,high,low', `comments` text COMMENT 'comments from the lab', `document_id` bigint(20) NOT NULL DEFAULT '0' COMMENT 'references documents.id if this result is a document', `result_status` varchar(31) NOT NULL DEFAULT '' COMMENT 'preliminary, cannot be done, final, corrected, incomplete...etc.' ) ENGINE=InnoDB DEFAULT CHARSET=latin1;”

It is noted that the tables regarding the MEDICAL SESSIONS (forms, form_encounter) and the LABORATORY MEASUREMENT UNITS (list_options) are omitted because their fields do not concern either the criteria or the outcome of the queries according to the criteria for obtaining the appropriate sample. The icd10_dx_order_code (ICD-10 ENCODING) table may be used to create criteria as a drop-down list.

Patient recruitment and prototype design

The following SQL code is executed to create the view from which the data for patient recruitment will be extracted (Table 5).

**Table 5:** View of patient extraction data.

**Figure 6:**
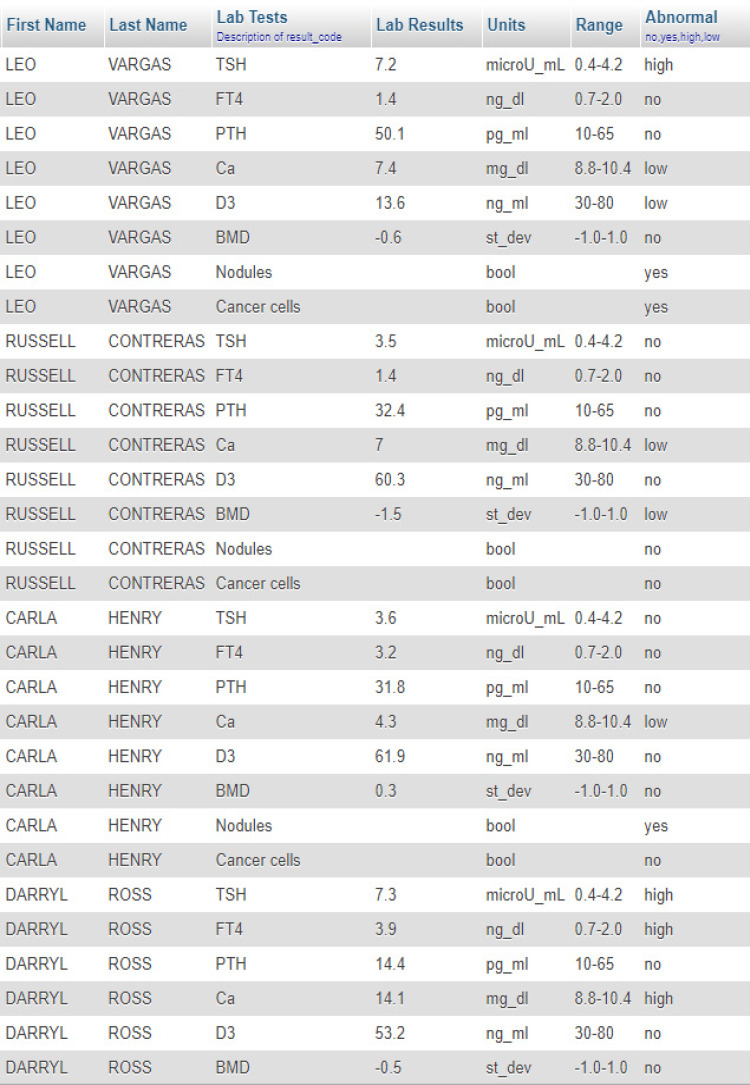
Screenshot of the patient “view”.

CREATE VIEW vw_PatientRecruitment
AS SELECT p.fname AS FirstName, p.lname AS LastName, pres.result_text AS LabTests, pres.result AS LabResults, pres.units AS Units, pres.range AS RangeOfValues, pres.abnormal AS Abnormal FROM patient_data p INNER JOIN procedure_order po ON p.pid = po.patient_id INNER JOIN procedure_report prep ON po.procedure_order_id = prep.procedure_order_id INNER JOIN procedure_result pres ON prep.procedure_report_id = pres.procedure_report_id;

The use of views is recommended for security reasons for the OpenEMR database and to avoid potential damage. The following is an instance of the view (Figure 6). For simplicity and without loss of information, the design of the form that will serve us results from the view based on selection criteria takes place on a stand-alone workstation using a portable USB web server. Thus, for the aforementioned view, the following PHP code is applied and serves as a static web page. Initially, a browser connection is created (Table 6). Code was obtained and adapted from OpenEMR [20], under the GNU General Public License (GNU GPL).

**Table 6:** The static webpage. Code obtained and adapted from OpenEMR [20], under the GNU General Public License (GNU GPL).

vw_connection.php
“”

The following is the main page code in which the criterion for patient selection is entered (Table 7). Code was obtained and adapted from OpenEMR [20], under the GNU General Public License (GNU GPL).

**Table 7:** Patient selection input. Code was obtained and adapted from OpenEMR [20], under the GNU General Public License (GNU GPL).

index.php
“ Patients recruitment Search input Please enter a lab test Search close(); ?> ”

**Figure 7:**
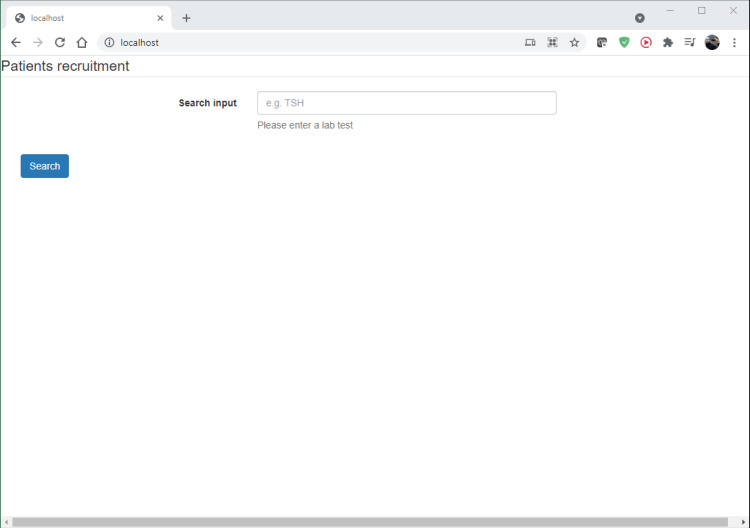
Screenshot of the static webpage.

After the code execution, the homepage would appear as in Figure 7.

The user enters, as a selection criterion, the lab test in the "Search" input field. After pressing the "Search" button, the following code is executed (Table 8). Code obtained and adapted from OpenEMR [20] under the GNU General Public License (GNU GPL).

**Table 8:** Code execution. Code obtained and adapted from OpenEMR [20] under the GNU General Public License (GNU GPL).

search_criteria.php
Testing criteria "; $sql = "SELECT FirstName, LastName, LabTests, LabResults, Units, RangeOfValues, Abnormal FROM vw_patientrecruitment WHERE LabTests LIKE '%" . $keywordfromlabtest . "%'"; $result = $mysqli->query($sql); if ($result->num_rows > 0) { // output data of each row while($row = $result->fetch_assoc()) { echo $row["FirstName"]. " " . $row["LastName"]. " " . $row["LabTests"]. " " . $row["LabResults"]. " " . $row["Units"]. " " . $row["RangeOfValues"] ." " . $row["RangeOfValues"] ." "; } } else { echo "0 results"; } ?> Return to the main page

**Figure 8:**
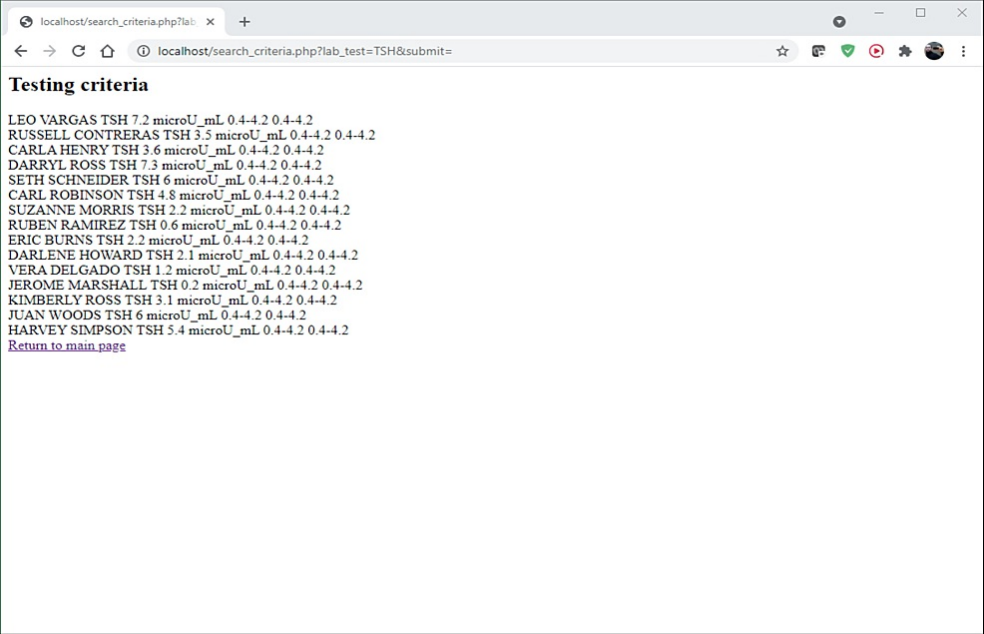
Screenshot of search results.

After executing the code, a new page with the results is displayed according to the lab test criterion (Figure 8). Return to the home page by clicking the "Return to main page" link.

## Discussion

In the present work, we have used open-source software and development tools to create an EMR database, emphasizing endocrinological conditions. We have verified that free and open-source software offers integrated solutions that can meet not only current needs, such as EMR, health unit administration, and patient recruitment but also potential needs that could arise in the immediate or distant future. Thus, the objective of the present study was to demonstrate that free and open-source software facilitates patient recruitment. For that purpose, we designed a prototype using free and open-source software. The appropriate software selection was based on quality criteria and findings from other studies. After installation and the required parameterization, the procured database was populated with the required data.

We relied on OpenEMR to design the patient recruitment prototype, based on both the enlisted tools in Table 3 and the proposal of Purkayastha S et al. [24]. After installing and commissioning the necessary software for our study, we created the appropriate control scenarios. To avoid the violation of sensitive personal data, the solution of creating dummy patients was preferred. Patient design and creation consider some specific features. Patients in the present study experienced diseases associated with the thyroid gland. The focus was given to three types of thyroid gland diseases: hypothyroidism, secondary hyperparathyroidism, and nodular goiter. For each disease, it is examined whether the values of the indicators are affected by gender, age, and weight. Accordingly, groups of patients are created based on the values that indicate pathological or physiological conditions. We aim to suggest an appropriate model for reliable patient recruitment according to the appropriate decision-making criteria.

A variety of work in the field of patient recruitment software has been conducted. Openness is a vital characteristic of these works, either in the form of open-source software or the form of open standards.

In the present work, we have used open source developing tools, including MySQL database, phpMyAdmin database front-end, OpenEMR integrated health information system, and Notepad++ editor. Our proposed prototype applies one selection criterion, which will be expanded in our ongoing work. Moreover, the selection criteria will not be entered as text, but rather selection will be made from drop-down menus. In addition, the proposed database will integrate more diseases and conditions. It is probable that such a solution would greatly help in the case of a plethora of health and public health incidents, for example, the present COVID-19 pandemic. For instance, such a database would facilitate the selection of patients with specific vaccine side effects. Various works in the field of patient recruitment software have been conducted. Openness is a key characteristic of these works, either in the form of open-source software or the form of open standards. In general, they have shown that it is possible to support the process of patient recruitment with information systems based on openness. However, they differ from each other in the following approaching methods. These methods address issues such as a) the type of utilized data. Some approaches make use of retrospective static data stored in data warehouses. Others are based on dynamically changing real-time data. Additionally, data heterogeneity plays a key role, such as b) deploying health information systems and software. Some approaches utilize already existing health information systems and software. Others propose the design of new systems according to specific needs and emerging requirements and c) emphasize the medical condition. Some approaches propose solutions emphasizing specific diseases. Others propose horizontally generic solutions to address various diseases and medical conditions.

With the above-mentioned text in mind, we present the following works chronologically by year. In their study, Stell A et al. (2008) outline the solution that has been implemented in the VOTES project. VOTES stands for Virtual Organisations for Trials and Epidemiological Studies. It was a pilot project to examine the implementation of a solution that combined distributed clinical data in real-time to facilitate processes such as patient recruitment. They utilized the data federation paradigm and grid technology to bring together the data from the clinical centers at several academic institutions. Data heterogeneity and security were two main challenges to address [26].

Weber S et al. (2010) described the implementation of event stream processing (ESP) on the STRIDE platform to address the challenge of real-time notification of patients presenting to the ED who might meet eligibility criteria for patient recruitment. STRIDE stands for Stanford Translational Research Integrated Database Environment. The implemented system used Esper [27], an open-source ESP/complex event processing (CEP) software package, along with an HL7 feed. The described system monitored real-time clinical data feeds using a pure ESP approach without reference to an existing database. It was employed for the automatic identification of eligible patients based on a combination of temporally related clinical criteria [28].

Ferranti JM et al. (2012) presented Duke Medicine's approach. To address the failure of patient recruitment, Duke Medicine implemented a hybrid solution, the Duke Integrated Subject Cohort and Enrolment Research Network (DISCERN). DISCERN combined both retrospective clinical data and clinical events contained in prospective HL7 messages. This approach aimed to immediately alert study personnel of potentially eligible patients. The DISCERN approach was employed in use cases for patient recruitment involving umbilical-cord blood collection, human papillomavirus vaccine study, and pediatric asthma study [29].

Damen D et al. (2013) introduced a framework for assisted patient recruitment called Platform for Assisted Semantic clinical Trial ELigibility (PASTEL). The PASTEL platform is developed on a semantic representation framework using Topic Maps that allows for formal representations of study protocols and reuse of eligibility criteria within and across institutions. The platform consists of (1) a web-based editor for the creation of ontology-based clinical trial representations, (2) a patient evaluator that connects to structured and unstructured hospital data sources to determine eligibility for a clinical trial, and (3) a web-based analytics module for reviewing evaluation results [30].

In their project, Trinczek B et al. (2014) designed and implemented a generic, thus portable, and scalable architecture for the software-based Project Recruitment System (PRS). This system was compatible with most of the available German Health Information System (HIS) environments. According to the authors, as HISs from different vendors varied in their functionality and customization, it was not guaranteed that these implementations could be adopted by other hospitals. This raised the challenge of finding a generic, scalable solution and implementation options for PRSs [31].

To support their proposition that an information dashboard can be a valuable tool to improve the conduct of clinical trials, Mattingly WA et al. (2015) designed and implemented a visualization dashboard for managing multi-site clinical trial enrollment in two pneumonia studies. This dashboard could be implemented as an add-on to existing systems or deployed as a separate web service using available data sources [32].

As a member of the King's Clinical Trials Unit, Markham S (2016) developed an initiative to create an online clinical trial recruitment portal/information hub for the National Institute for Health Research (NHIR) Mental Health Biomedical Research Centre (BRC). The primary purpose of this initiative was to promote patient and public awareness of and interest in participating in clinical trials. The author was a mental health service user who entered service user involvement work as part of her recovery from a severe suicide attempt. She applied to become a member of the BRC's Service User Advisory Group (SUAG), which is a group of mental health service users and carers, each of whom have academic experience [33].

Lindsay J et al. (2017) developed MatchMiner [34]. MatchMiner is an open-source computational platform for matching patient-specific genomic profiles to precision cancer medicine clinical trials. The authors stated that given the complexity of tumor profiling and the rapidly changing multi-site nature of genome-driven clinical trials, open-source software is the most efficient, scalable, and economical option for patient recruitment in clinical trials [35].

Augustinov G and Duftschmid G (2019) state that automatic comparison of routinely collected EHR data with trial eligibility criteria can accelerate patient recruitment. Their work aims to support this statement on the Austrian nationwide EHR system ELGA (German acronym for "Electronic Health Record"). Using the open source tool ART-DÉCOR [36], the authors tried to map a reference list of 150 common eligibility criteria specified in the Electronic Health Records for Clinical Research (EHR4CR) project to the HL7 CDA templates that describe the structure of ELGA document types. Comparing their results with similar work, they conclude that ELGA could be a useful component for the automatic identification of eligible patients [37].

In their work, Kotoulas A et al. (2019) describe the design and virtual implementation of a web-based prototype biomedical registry in Greece. The approach was employed in use cases for patient registration involving colorectal cancer studies. The system represents an eGovernment framework proposal for the central storage of patients' information. The designed system is based on free, open-source software and is implemented virtually in a local host environment [38].

Reinecke I et al. (2020) presented the design of a support system based on open-source solutions and open standards. More specifically, the Medical Information in Research and Care in University Medicine (MIRACUM) consortium designed a Clinical Trials Recruitment Support System (CTRSS) to support data-driven patient recruitment for clinical trials. The design of the prototype included both open-source solutions (OMOP CDM, Atlas) and open standards for interoperability (FHIR). The aim of the prototype was to implement a patient screening list of potential candidates for a clinical study. Their work showed the modular structure and functionality of the prototype building the foundation for the practical implementation of the CTRSS and, at the same time, demonstrating the use of open-source solutions and standards for the design of clinical support systems [39].

Little JA et al. (2020) outline the efforts of Programa de Estudio y Control de Enfermedades Tropicales (PECET) in the treatment of cutaneous leishmaniasis in rural Colombia. PECET is a multidisciplinary tropical medicine research group based at the University of Antioquia, Colombia. PECET conducted the above-mentioned treatment using the OpenMRS database. In addition, their work describes the potential use of mobile digital tools to assist PECET with patient recruitment and retention in clinical trials [40].

Kotoulas A and Koutsouris D-D (2020) developed virtual COVID-19 datasets to demonstrate a generic COVID-19 patient recruitment system in Greece. In addition, they developed a custom interactive map using an open-source Javascript library to display valuable information for clinical trial researchers and crisis managers [41]. Banach A et al. (2021) outlined the development of a fully automated patient recruitment solution called APERITIF. APERITIF can identify the number of eligible patients based on free-text eligibility criteria, considering the Medical Information Initiative (MII) core data set and based on the FHIR standard. This approach extracts medical and demographic data from free-text eligibility criteria of a study using Natural Language Processing (NLP) methods [42].

Advantages and disadvantages of using EMR

The use of EMR is up-to-date, a real need, since the fast and direct transmission of information is of paramount importance. Thus, the EMR, besides its role in information processing, plays a crucial role in improving patients' quality of life. There are numerous advantages of using EMR, also reported in the literature [43]. Such advantages include: a) the simplicity of entering, searching and recording data, i.e., the quick and immediate access to the medical information whenever needed, b) the fast and immediate entry of laboratory test data, c) the ease of finding data at the local level but also in patients' file systems, d) the assistance in the diagnosis through access to a knowledge-based system, e) better analysis of patient data, f) the possibility of statistical processing and analysis of data in the context of a statistical study, g) the evaluation of the therapeutic effect based on the comparison with similar cases and their treatment so far, h) the assistance in the diagnosis/treatment of patients in remote areas through telemedicine, i) improving of health services quality provided to the general public, j) the continuous and better communication on issues related to public health, k) the reduction of healthcare costs and l) the EMR's contribution to research and development is very important due to the fact that its database enables the monitoring of diseases and the provision of preventive measures when needed, and last but not least, m) integrating an EMR in the clinical praxis will reduce healthcare costs significantly.

In previous work, we reported that the implementation of the EMR may be based on existing manual procedures, which, when improved, could prove helpful towards the development of patient databases for clinical studies [13]. Patient recruitment is closely connected to the EMR. On the academic and research level, the use of EMR is an essential tool for selecting patients for clinical trials. Also, there is a close relationship between EMR and the decision-support system (DSS). In that sense, an endocrinology unit (as this was the example of our previous work) can benefit from the integration effort of the aforementioned systems. The area of free and open-source software offers integrated solutions that can meet the needs of an EHR, patient recruitment, decision-making in a medical unit (i.e., an endocrinology unit), and future needs that may arise.

Future perspectives

In the present work, we have used open source developing tools, including MySQL database, phpMyAdmin database front-end, OpenEMR integrated health information system, and Notepad++ editor. Our proposed prototype applies one selection criterion, which will be expanded in our ongoing work. Moreover, the selection criteria will not be entered as text. But instead, selection will be made from drop-down menus. In addition, the proposed database will integrate more diseases and conditions. It is probable that such a solution would greatly help in the case of a plethora of health and public health incidents, such as the present COVID-19 pandemic. For instance, such a database would facilitate the selection of patients with specific vaccine side effects. In our future endeavors, we will expand our efforts and implement an EMR, putting it into function in an endocrinology unit's clinical setting in Greece.

Study limitations

In order to show the full operability of such an electronic medical system, large amounts of data are required. Moreover, the additive complexity of medical records can lead to data handling problems, which can be faced only by adding multiple records to such a database. Thus, the following steps would include enrichment of the created database with more data to check for its operability. Further, emphasis could be placed on the functionalities and how the final information is presented based on the selection criteria. For instance, the application could be enriched by displaying the data in tabulated form. At the same time, it would be essential and helpful to use appropriate graphs to present the results. Also, the porting of the application to mobile devices would provide usability and flexibility to users whose work is characterized by frequent commuting.

## Conclusions

The use of EMR is an essential tool for the selection of patients for clinical trials. Also, there is a close relationship between EMR and a DSS. In that sense, as mentioned earlier, an endocrinology unit (as this was the example of our previous work) can benefit from the integration effort of the systems. The area of free and open-source software offers integrated solutions that can meet the needs of an EHR, patient recruitment, decision-making in a medical unit (i.e., an endocrinology unit), and future needs that may arise.
